# Characteristics of Physicians Excluded From US Medicare and State Public Insurance Programs for Fraud, Health Crimes, or Unlawful Prescribing of Controlled Substances

**DOI:** 10.1001/jamanetworkopen.2018.5805

**Published:** 2018-12-14

**Authors:** Alice Chen, Daniel M. Blumenthal, Anupam B. Jena

**Affiliations:** 1 Sol Price School of Public Policy, University of Southern California, Los Angeles; 2 Leonard D. Schaeffer Center for Health Policy and Economics, University of Southern California, Los Angeles; 3 Cardiology Division, Department of Medicine, Massachusetts General Hospital, Boston; 4 Harvard Medical School, Boston, Massachusetts; 5 Devoted Health Inc, Waltham, Massachusetts; 6 Department of Health Care Policy, Harvard Medical School, Boston, Massachusetts; 7 Massachusetts General Hospital, Boston; 8 National Bureau of Economic Research, Cambridge, Massachusetts

## Abstract

**Importance:**

Each year, billions of dollars are wasted owing to health care fraud, waste, and abuse. Efforts to detect fraud have been increasing, yet we have little information about physicians who have been excluded from Medicare and state public insurance programs for fraud, health crimes, or the unlawful prescribing of controlled substances.

**Objective:**

To examine the characteristics of physicians excluded from Medicare and state public insurance programs for fraud, health crimes, or unlawful prescribing of controlled substances.

**Design, Setting, and Participants:**

This cross-sectional study considered all physicians excluded from Medicare and state public insurance programs between 2007 and 2017. The study matched exclusion data to a comprehensive, cross-sectional database of US physicians assembled by Doximity, an online networking service for US physicians. The share of physicians excluded in each state was examined and linear trends of exclusions over time were estimated. Using physician-level multivariable logistic regression models, exclusions (binary variable) were assessed as a function of physician characteristics.

**Main Outcomes and Measures:**

Exclusions for fraud, health crimes (defined legally as criminal penalties for acts involving federal health care programs), and substance abuse; and physician characteristics, including age, sex, allopathic vs osteopathic degree, medical school attended, ranking of that medical school, medical school faculty affiliation, practice state, practice location, and specialty.

**Results:**

Between 2007 and 2017, 2222 physicians (0.29%) were temporarily or permanently excluded from Medicare and state public insurance programs. Fraud, health crimes, and substance abuse exclusions increased, on average, 20% per year (equivalent to 48 [95% CI, 40.4-56.0] convictions/year from a base of 236 convictions in 2007 to 670 convictions in 2017 [an increase of approximately 200% from 2007 to 2017]). Exclusion rates were highest in the West and Southeast. West Virginia had the highest exclusion rate, with 5.77 exclusions per 1000 physicians (32 exclusions among 5720 physicians), while Montana had 0 exclusions during this period. Male physicians, physicians with osteopathic training, older physicians, and physicians in specific specialties (eg, family medicine, psychiatry, internal medicine, anesthesiology, surgery, and obstetrics/gynecology) were more likely to be excluded.

**Conclusions and Relevance:**

The number of physicians excluded from participation in Medicare and state public insurance reimbursement owing to fraud, waste, and abuse increased between 2007 and 2017. Several physician characteristics, including being a male, older age, and osteopathic training, were significantly and positively associated with exclusion. Our results highlight the potential value of using physician characteristics in conjunction with information on medical claims filed by physicians to help identify adverse physician behavior.

## Key Points

**Question:**

What were the characteristics of physicians who have been excluded from US Medicare and state public insurance programs?

**Findings:**

In this cross-sectional study assessing all physician exclusions from 2007 to 2017, the number of physician exclusions grew by 20% per year (equivalent to 48 additional exclusions per year) to encompass approximately 0.3% of US physicians in 2017. Exclusions were more common in the West and Southeast census regions, and male physicians, physicians with osteopathic training, older physicians, and physicians in specific specialties (eg, family medicine, psychiatry, internal medicine, anesthesiology, surgery, and obstetrics/gynecology) were more likely to be excluded.

**Meaning:**

The likelihood of exclusion varied across regions and with physician demographics and specialty.

## Teaser

This cross-sectional study examines the characteristics of physicians excluded from participation in US Medicare and state public health insurance plans between 2007 and 2017 owing to fraud, health crimes, or unlawful prescribing of controlled substances.

## Introduction

Limited information exists on the characteristics of US physicians who have been excluded from Medicare and state public insurance programs for convictions of health care fraud, crimes related to health care delivery, or substance abuse. Common fraud schemes include billing for services not rendered, filing duplicate claims (including the unbundling of bundled services), and misrepresenting dates and locations where services were provided. Health crimes involve the provision of medically unnecessary procedures, illegal patient admittance and retention practices, the making of false statements (including physician medical identify theft), and the gross violation of professionally recognized standards of care. Substance abuse exclusions result from the illegal distributing, prescribing, or dispensing of controlled substances such as prescription opioids and surgical anesthetics.

According to the Institute of Medicine, fraud, waste, and abuse in 2009 reached $750 billion (or 28% of total health care spending) with fraud alone constituting $75 billion (or 3% of total health care spending).^1^ Other sources, including the Federal Bureau of Investigation, suggest that fraudulent billings have ranged up to $260 billion in 2010 (or 10% of total health care spending).^2,3^ More recently, policymakers have taken several steps to reduce health care fraud, waste, and abuse, including establishing an interagency Medicare Fraud Strike Force in 2007 and laying forth provisions in the Patient Protection and Affordable Care Act (2010) and Small Business Jobs Act (2010) to prevent fraud and enable the prosecution of health care professionals who engage in fraudulent activities.^4,5,6^

Previous studies of physician fraud and other exclusions from Medicare rely on older data^7,8,9^ and do not include sufficient comparisons of the characteristics of excluded and nonexcluded physicians.^7,8,9,10,11^ Published studies of board disciplined physicians were limited to case studies from specific states.^8,10^ More contemporary, comprehensive data on the number of physicians excluded from reimbursement by Medicare and state public insurance programs owing to concerns about fraud, waste, and abuse and the types of physicians who are more likely to be excluded would be helpful for understanding the scale of potentially wasteful service delivery in the United States and the success of ongoing efforts to deter, prevent, and identify health care fraud. Therefore, we evaluated trends in rates and geographical distribution of physician exclusions, and assessed the characteristics of excluded physicians using a contemporary, nationally representative database of physicians excluded from publicly funded health care programs for offenses related to medical fraud, abuse of controlled substances, and health care crimes.

## Methods

### Data Sources and Study Sample

We identified all physicians who were excluded from Medicare and state public insurance programs from 2007 to 2017 using data from the US Office of Inspector General, which has the right to exclude individuals and entities from public insurance participation for reasons specified in Section 1128 of the Social Security Act. Physicians may be excluded for several reasons, including fraud (codes 1128a3, 1128b[1]-[2], or 1128b[4]-[7]), unlawful prescribing or dispensing of controlled substances (codes 1128a4 or 1128b3), or health crime convictions (codes 1128a1 or 1128a2) related to the delivery of services under Medicare, Medicaid, the State Children’s Health Insurance Program, or other state health care programs.

To obtain personal and professional characteristics for excluded physicians, we used each physician’s unique national provider identifier to match them to their profile in Doximity, an online networking service for US physicians. Doximity maintains a comprehensive database of licensed US physicians, and it gathers and continuously updates several pieces of personal and professional information about each physician in the database. Data from the Doximity database have been used in previous studies.^12,13,14,15,16^ Doximity obtains data on physicians’ personal and professional characteristics via multiple sources and data partnerships, including the National Plan and Provider Enumeration System, the American Board of Medical Specialties, state medical boards, and collaborating hospitals and medical schools. Previous studies have validated data for a random sample of physicians in the Doximity database by using manual audits.^15,16^ We were able to match 86% of physicians in the exclusions database to their profile in the Doximity database.

This study was considered to not involve human subjects research by the institutional review board at Harvard Medical School. This study followed the Strengthening the Reporting of Observational Studies in Epidemiology (STROBE) reporting guideline for reporting cross-sectional studies.^17^

### Physician Characteristics

The Doximity database contains information on several physician characteristics, including physicians’ sex, age, type of medical degree (osteopathic vs allopathic medical degree), clinical specialty, having a faculty appointment at a US medical school, practice state, degree of rurality of practice location (assigned as urban vs rural based on the US Department of Agriculture’s Rural-Urban Continuum Codes and practice zip code), medical school attended (including international medical graduates [IMGs]), and ranking of the medical school attended according to *US News & World Report* 2013 rankings.

### Statistical Analysis

First, we evaluated how the universe of physician exclusions from 2007 to 2017 evolved across geography and time. We calculated rates of geographical exclusions by state and region (Northeast, Southeast, West, and South) and used linear regressions to identify how rates of exclusions have changed over time. Rates were presented as the number of excluded physicians per 1000 physicians in a given geographical area.

Next, we evaluated for associations between physician characteristics and exclusion from participation in Medicare or state public insurance programs. Physician characteristics included indicator variables for IMGs (binary); doctor of medicine vs doctor of osteopathic medicine degree (binary); graduating from a top 20–ranked medical school according to *US News & World Report* (binary); having a faculty appointment at a US medical school (binary); practicing in an urban location (binary); being male vs female (binary); age, based on 5 categories (ages ≤34 years, 35-44 years, 45-54 years, 55-64 years, and ≥65 years); and 16 specialty categories (anesthesiology, cardiology, emergency medicine, family medicine, gastroenterology, internal medicine, neurology, obstetrics and gynecology, orthopedic surgery, pathology, pediatrics, psychiatry, radiology, surgery, surgery subspecialties, and all other specialties).

We estimated physician-level, multivariable logistic regression models of exclusion from participation in Medicare or state public insurance programs (binary variable) as a function of the above physician characteristics. The 95% CI around reported estimates reflects 0.025 in each tail or *P* ≤ .05. Stata statistical software, version 15.1 (StataCorp) was used for analysis.

## Results

### Characteristics of Exclusions

**Figure 1.**
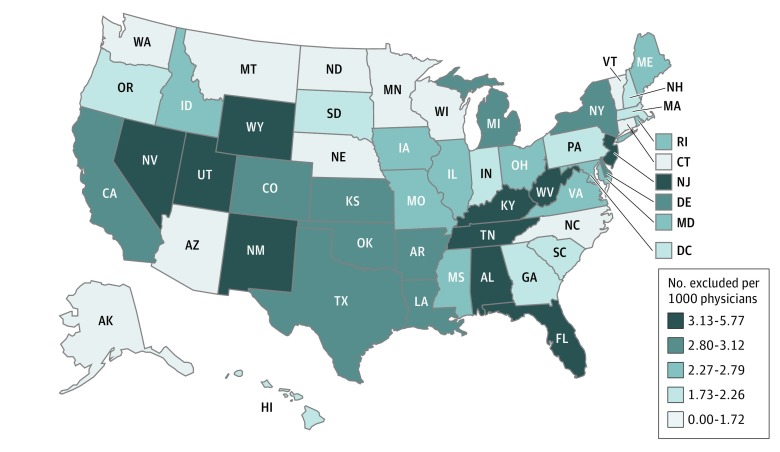
Number Excluded per 1000 Physicians, by State States were divided into 5 quintiles based on the number of physicians excluded per 1000 physicians. West Virginia had the highest physician exclusion rate with 5.77 exclusions per 1000 physicians. Montana had the lowest exclusion rate (ie, 0 exclusions) during this period.

Physicians in the West and Southeast were most likely to be excluded for fraud, substance abuse, or health crimes (Figure 1). Although California (n = 324), New York (n = 252), Florida (n = 247), and Texas (n = 184) had the highest absolute counts of excluded physicians from 2007 to 2017, they also had the largest physician populations. When considering the rate of physician exclusions per 1000 physicians, only Florida remained in the highest category of exclusion rates. West Virginia had the highest exclusion rate, with 5.77 exclusions per 1000 physicians (32 exclusions among 5720 physicians), while Montana had 0 exclusions during this period.

**Figure 2.**
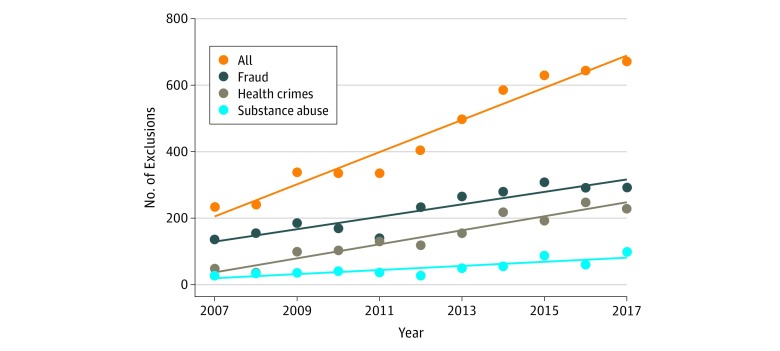
Physician Exclusion Time Trends The figure shows number of physicians, by exclusion category and year, and linear time trends of physician exclusions in each category.

Total physician exclusions increased 20% per year, on average, between 2007 and 2017 (an increase of 48.22 [95% CI, 40.41-56.03] exclusions/year, from a base level of 236 exclusions in 2007; *P* < .001). Yearly growth in the number of excluded physicians was particularly large after 2011 (Figure 2). Exclusions for fraud (which increased 14% per year or 18.77 [95% CI, 12.61-24.94] exclusions/year, from a base level of 139 exclusions in 2007; *P* < .001) and health care crimes (which increased 46% per year or 23.26 [95% CI, 18.97-27.56] exclusions/year, from a base of 67 exclusions in 2007; *P* < .001) accounted for the majority of the absolute increase in physician exclusions between 2007 and 2017. Exclusions related to the unlawful prescribing of controlled substances constituted a smaller share of total exclusions, and exclusions for this category increased 21% per year, on average (6.07 [95% CI, 3.10-9.05] exclusions/year, from a base of 29 exclusions in 2007; *P* = .001).

### Physician Characteristics

Between 2007 and 2017, 2222 physicians (0.29%) were temporarily or permanently excluded from Medicare and state public insurance programs. In unadjusted odds ratio (OR) analysis, IMGs (1.42; 95% CI, 1.29-1.55), male physicians (1.70; 95% CI, 1.53-1.88), and older physicians (4.63; 95% CI, 3.57-6.01)—in each of the age categories relative to physicians younger than 35 years—were more likely to be excluded (Table 1). Exclusions were most common in family medicine (n = 398) and psychiatry (n = 213) and least common in cardiology (n = 49) and radiology (n = 54). Exclusions were less common among doctors of medicine (relative to doctors of osteopathic medicine), graduates of top 20 medical schools (n = 214; as defined by *US News & World Report* 2013 rankings), physicians with faculty appointments at US medical schools (n = 117), and physicians practicing in urban locations (n = 2177).

**Table 1.** Characteristics of Physicians Excluded From Medicare and State Health Insurance Programs

Physician Characteristic	No. Excluded	No. Not Excluded	Unadjusted OR (95% CI)	Adjusted OR (95% CI)^a^
International medical graduates	630	169 814	1.42 (1.29-1.55)	1.30 (1.18-1.44)
MD degree	2105	743 709	0.79 (0.65-0.95)	0.76 (0.63-0.92)
Attended top 20 medical school^b^	214	100 020	0.72 (0.62-0.83)	0.86 (0.74-1.00)
Faculty member at US medical school	117	92 261	0.41 (0.34-0.50)	0.48 (0.40-0.58)
Urban location	2177	766 591	0.59 (0.44-0.79)	0.84 (0.62-1.13)
Gender				
Male	1750	532 578	1.70 (1.53-1.88)	1.52 (1.37-1.69)
Female	472	223 291	1 [Reference]	1 [Reference]
Age, y				
≤34	63	79 360	1 [Reference]	1 [Reference]
35-44	302	182 289	2.09 (1.60-2.74)	2.10 (1.60-2.75)
45-54	557	180 552	3.89 (2.99-5.04)	3.69 (2.84-4.79)
55-64	720	175 617	5.17 (4.00-6.69)	4.70 (3.63-6.10)
≥65	580	158 051	4.63 (3.57-6.01)	4.05 (3.11-5.26)
Specialty				
Anesthesiology	147	44 937	1.71 (1.17-2.50)	1.67 (1.14-2.44)
Cardiology	49	27 383	0.94 (0.60-1.46)	0.84 (0.54-1.31)
Emergency medicine	116	40 961	1.48 (1.01-2.18)	1.55 (1.05-2.20)
Family medicine	398	91 083	2.29 (1.60-3.27)	2.21 (1.55-3.16)
Gastroenterology	27	14 731	0.96 (0.58-1.60)	0.87 (0.52-1.45)
Internal medicine	347	102 686	1.78 (1.24-2.54)	1.85 (1.29-2.64)
Neurology	56	16 840	1.74 (1.13-2.68)	1.82 (1.18-2.79)
Obstetrics and gynecology	142	42 577	1.75 (1.20-2.55)	1.86 (1.27-2.71)
Orthopedic surgery	56	26 966	1.09 (0.71-1.67)	1.05 (0.68-1.62)
Pathology	33	17 284	1.13 (0.79-1.63)	1.17 (0.81-1.68)
Pediatrics	115	73 283	0.82 (0.56-1.21)	1.01 (0.69-1.49)
Psychiatry	213	45 242	2.48 (1.72-3.58)	2.38 (1.65-3.43)
Radiology	54	37 932	0.75 (0.48-1.15)	0.76 (0.49-1.17)
Surgery	109	34 122	1.69 (1.14-2.49)	1.73 (1.17-2.55)
Surgical subspecialty	107	43 492	1.29 (0.87-1.90)	1.23 (0.83-1.82)
Other	253	102 739	1 [Reference]	1 [Reference]
Observations, No.	2222	775 869	778 091	778 091

Abbreviations: MD, doctor of medicine; OR, odds ratio.

^a^ Estimates were from a multivariable logistic regression of the probability of being excluded as a function of the listed physician characteristics.

^b^ Top 20 medical school according to *US News & World Report* 2013 medical school research rankings.

After multivariable adjustment, physicians who were male (adjusted OR, 1.52; 95% CI, 1.37-1.69; *P* < .001), older, had a doctor of osteopathic medicine degree, were IMGs (adjusted OR, 1.30; 95% CI, 1.18-1.44; *P* < .001), did not attend a top 20–ranked US medical school, and were not affiliated faculty at a medical school had higher adjusted odds of exclusion. The adjusted ORs of exclusion remained highest in family medicine (2.21; 95% CI, 1.55-3.16; *P* = .03) and psychiatry (2.38; 95% CI, 1.65-3.43; *P* < .001) and lowest in cardiology (0.84; 95% CI, 0.54-1.31; *P* = .44) and radiology (0.76; 95% CI, 0.49-1.17; *P* = .21). After multivariable adjustment, practicing medicine in an urban location was no longer associated with exclusion.

### Differences by Type of Exclusion

Certain physician characteristics—including being male, being older, and not having a faculty appointment at a US medical school—were associated with greater odds of exclusion independent of the reason for exclusion (Table 2).

**Table 2.** Characteristics of Physicians Excluded From Medicare and State Health Insurance Programs, by Type of Exclusion

Physician Characteristic	Adjusted OR (95% CI)^a^		
	Fraud	Health Crime	Substance Abuse
International medical graduates	0.95 (0.83-1.09)	1.62 (1.37-1.91)	1.34 (1.04-1.73)
Male	1.24 (1.09-1.42)	1.52 (1.26-1.83)	2.18 (1.59-2.99)
MD degree	0.88 (0.68-1.14)	0.80 (0.56-1.15)	0.54 (0.35-0.83)
Attended top 20 medical school^b^	0.97 (0.81-1.15)	0.66 (0.49-0.88)	0.81 (0.53-1.22)
Faculty member at US medical school	0.48 (0.38-0.61)	0.69 (0.52-0.93)	0.25 (0.12-0.50)
Urban location	0.73 (0.50-1.06)	1.17 (0.62-2.19)	0.51 (0.29-0.92)
Age, y			
≤34	1 [Reference]	1 [Reference]	1 [Reference]
35-44	1.89 (1.38-2.60)	1.93 (1.21-3.07)	4.42 (1.58-12.35)
45-54	2.92 (2.15-3.96)	3.36 (2.16-5.25)	6.94 (2.53-19.05)
55-64	3.75 (2.77-5.07)	3.95 (2.54-6.15)	10.94 (4.02-29.75)
≥65	3.43 (2.52-4.65)	3.79 (2.42-5.93)	9.59 (3.51-26.25)
Specialty			
Anesthesiology	1.69 (1.06-2.68)	1.13 (0.57-2.24)	1.24 (0.53-2.88)
Cardiology	0.67 (0.38-1.19)	0.68 (0.30-1.52)	0.82 (0.32-2.13)
Emergency medicine	1.35 (0.84-2.19)	1.31 (0.65-2.62)	1.18 (0.50-2.80)
Family medicine	1.70 (1.09-2.65)	2.20 (1.18-4.08)	1.72 (0.79-3.76)
Gastroenterology	0.90 (0.48-1.68)	1.00 (0.42-2.36)	0.29 (0.06-1.37)
Internal medicine	1.33 (0.85-2.08)	2.24 (1.21-4.14)	1.53 (0.70-3.34)
Neurology	1.63 (0.95-2.80)	1.84 (0.87-3.86)	1.67 (0.65-4.31)
Obstetrics and gynecology	1.76 (1.10-2.80)	1.70 (0.87-3.31)	0.92 (0.37-2.25)
Orthopedic surgery	1.09 (0.64-1.83)	0.99 (0.46-2.15)	0.57 (0.20-1.64)
Pathology	1.08 (0.69-1.68)	1.14 (0.61-2.14)	0.75 (0.33-1.69)
Pediatrics	1.05 (0.65-1.68)	0.98 (0.50-1.93)	0.45 (0.17-1.17)
Psychiatry	2.38 (1.52-3.73)	2.08 (1.09-3.95)	0.86 (0.36-2.09)
Radiology	0.63 (0.36-1.09)	0.76 (0.36-1.63)	0.32 (0.10-1.00)
Surgery	1.75 (1.09-2.81)	1.71 (0.87-3.37)	0.62 (0.23-1.68)
Surgical subspecialty	1.24 (0.77-2.00)	1.32 (0.67-2.60)	0.36 (0.12-1.02)
Other	1 [Reference]	1 [Reference]	1 [Reference]
Observations, No.	777 035	776 477	776 166

Abbreviations: MD, doctor of medicine; OR, odds ratio.

^a^ Estimates were from a multivariable logistic regression of the probability of being excluded as a function of the listed physician characteristics.

^b^ Top 20 medical school according to *US News & World Report* 2013 medical school research rankings.

For other physician characteristics, the strength of the association between the physician characteristic and the odds of exclusion differed by reason for exclusion. For example, IMGs had higher adjusted ORs of health crime (1.62; 95% CI, 1.37-1.91) and substance abuse (1.34; 95% CI, 1.04-1.73) exclusions, but not fraud (0.95; 95% CI, 0.83-1.09) exclusions. The adjusted ORs of exclusion for fraud and health crimes, but not substance abuse, were significantly associated with practicing family medicine (1.70; 95% CI, 1.09-2.65 and 2.20; 95% CI, 1.18-4.08, respectively) and psychiatry (2.38; 95% CI, 1.52-3.73 and 2.08; 95% CI, 1.09-3.95, respectively). The adjusted ORs for fraud, but not for health crimes or substance abuse, were higher for surgery (1.75; 95% CI, 1.09-2.81), anesthesiology (1.69; 95% CI, 1.06-2.68), and obstetrics and gynecology (1.76; 95% CI, 1.10-2.80) physicians. The adjusted ORs for health crimes were higher for internal medicine physicians (2.24; 95% CI, 1.21-4.14).

## Discussion

The study evaluated geographical and temporal trends in rates of physician exclusion from participation in federal and state public health insurance plans owing to potential fraud, waste, and abuse, and the relationship between several physician characteristics and exclusion. The study found that approximately 0.3% of US physicians were temporarily or permanently excluded from Medicare and state public insurance programs between 2007 and 2017 for fraud, unlawful prescribing of controlled substances, or health crimes. The number of excluded physicians increased on average, by 20% per year (48 additional exclusions/year) between 2007 and 2017. After multivariable adjustment, male sex, older age, graduating from an osteopathic medical school or being an IMG, not having a faculty appointment at a US medical school, and practicing family medicine, psychiatry, internal medicine, anesthesiology, surgery, and obstetrics and gynecology were significantly and positively associated with exclusion.

To our knowledge, this study represents the most comprehensive and contemporary effort to assess trends in physician exclusion from participation in public health insurance owing to fraud, waste, and abuse concerns, and physician characteristics associated with exclusion. This study found that the numbers of physicians excluded from participation in public health insurance increased by approximately 200% during a 10-year period (from 236 in 2007 to 670 in 2017).

There were several explanations for the observed increase in exclusions, and rates of identified health care fraud, waste, and abuse. First, this finding could be evidence that regulators, who have been aided by recent public policies targeting the reduction of fraud and waste, may be getting better at identifying perpetrators of fraudulent activity. The Affordable Care Act allocated $350 million (beginning in 2011) to the US Department of Health and Human Services’ Health Care Fraud and Abuse Account and increased sanctions on questionable providers, including allowing state Medicaid programs to halt payments, requiring that Medicare overpayments be returned within 60 days (instead of 3 years), and increasing the penalty for a false claim from $10 000 per claim to $50 000 per claim.^18,19^ In addition, the Small Business Jobs Act of 2010 committed Medicare to a 5-year time table to develop and apply predictive analytics to prevent fraud.^5,20^ The Centers for Medicare & Medicaid Services has used predictive analytics to detect improper billing since July 2011.^5,6^ This combination of increased funding for identifying and preventing health care fraud, harsher sanctions for potential perpetrators of fraud, and new tools for identifying fraud may have helped regulators to identify greater numbers of physicians engaging in fraudulent activity.

In addition, the growth in physician exclusions could also be due, at least in part, to growth in the total number of US physicians participating in public insurance. Enrollment in public insurance programs increased significantly after the passage of the Affordable Care Act; enrollment in any government health insurance plan increased by 12.6% total from 2013 to 2017, higher than the 7.9% increase into private insurance.^21^ In parallel, the number of physicians treating patients with public insurance has also expanded. Thus, it is possible that at least some of the increase in physician exclusions was associated with the expansion of the total pool of physicians that Medicare and state insurance programs were monitoring for evidence of fraud, waste, and abuse. We cannot exclude the possibility that the increase in physician exclusions reflects a rise in fraudulent and untoward practices by US physicians. However, we are unaware of any published data that support this potential explanation.

We found that physician exclusions were more common in certain states in the West and Southeast. Many of these regions had Medicare Fraud Strike Force Teams, which were established in “hot spots” of unexplained high Medicare billing levels (Florida, California, Michigan, Texas, New York, Louisiana, Florida, and Illinois as of 2017).^4^ They also corresponded to states with high levels of Medicare waste per beneficiary, calculated as Medicare overpayments for inaccurate bills or high levels of risk-adjusted, total Medicare spending per episode of care.^22,23^ For example, the high exclusion rate states of California in the West; Texas, Oklahoma, and Arkansas in the South; and New Jersey and Delaware in the East had levels of Medicare waste that ranked in the top 20% nationally. New Jersey, Florida, and Louisiana had the highest levels of per capita Medicare spending based on standardized spending measures that removed geographical differences in payment as a source of variation.^24^

Exclusion was more common among male physicians, physicians with osteopathic training, older physicians, and physicians in specific specialties (eg, family medicine, psychiatry, internal medicine, anesthesiology, surgery, and obstetrics/gynecology). While the study identified several personal and professional characteristics of physicians that were associated with greater odds of exclusion from public insurance, the magnitude of these associations was, for the most part, modest. However, the higher odds of exclusion for fraud and health crime exclusions observed among family medicine physicians and psychiatrists departed from this trend. One potential explanation for this finding is that fraud is easier to carry out when the risk of malpractice suits is particularly low, as they are in the fields of family medicine and psychiatry.^25^ Notably, these specialties are not statistically significantly associated with higher rates of substance abuse exclusions, with the magnitude of the OR being less than 1 for psychiatrists.

Our results highlight the potential value of using physician characteristics, in conjunction with information on medical claims filed by physicians, to help identify adverse physician behavior. In their predictive models, Centers for Medicare & Medicaid Services already uses fee-for-service claims data to identify clinician behaviors that warrant administrative actions.^26^ However, some of these models have high false-positive rates^27^ and have led regulators to invest significant time and resources into investigations of physicians who are not engaged in untoward activities. Therefore, improving the sensitivity and specificity of these predictive models could increase the efficiency with which regulators allocate limited investigation and enforcement resources. In light of differences in the adjusted ORs of exclusion that were associated with specific physician characteristics, identifying outliers within these characteristics may help identify patterns that are actually aberrant. For example, these models may be improved by controlling for geographical variations in fraud, specialty-specific variation in behavior, and age differences, gender differences, and training differences that may be associated with practice- or patient-based differences.

### Limitations

This study had several limitations. First, the cross-sectional study design limits causal inference. However, determining associations between physician characteristics and fraudulent behavior is an essential first step in identifying characteristics that may help to potentially associate which physicians are more or less likely to engage in fraudulent activities. Second, this study only focused on physicians who have been identified as fraudulent. These exclusions typically represent those who have committed egregious acts of fraud, health crime, or substance abuse; since its inception in March 2007, the Medicare Fraud Strike Force has charged more than 4000 defendants who collectively have falsely billed the Medicare program more than $14 billion.^28^ The characteristics of those committing lesser acts of fraud may be different than those observed in this research. Third, we have limited data on practice- and patient-specific characteristics that may shed light on why certain physician characteristics were associated with higher exclusion rates. Fourth, we cannot rule out confounding factors owing to unmeasured variables.

## Conclusions

In this study, we found that the number of physicians excluded from participating in public health insurance has grown substantially over time and that excluded physicians were concentrated in specific regions of the United States. In addition, the odds of being excluded were significantly higher among physicians who were older, were male, graduated from osteopathic medical schools, lacked a medical school faculty affiliation, and practiced family medicine, psychiatry, obstetrics and gynecology, or surgery. Identifying these associations lays the foundation for further studies to illuminate the mechanisms underlying these associations and their potential for improving predictive models.
